# Cachexia in mice blunts improved cognitive flexibility induced by calorie restriction

**DOI:** 10.1016/j.bbi.2025.106123

**Published:** 2025-09-27

**Authors:** Kyna Conn, Laura K. Milton, Alyssa Teoh, Priscila T. Levi, Kelly L. Walton, Zane B. Andrews, Claire J. Foldi, Sarah H. Lockie

**Affiliations:** a Monash Biomedicine Discovery Institute and Department of Physiology, Monash University, Clayton, Victoria 3800, Australia; b School of Biomedical Sciences, University of Queensland, St. Lucia, Qld, Australia

**Keywords:** Cancer cachexia, Behaviour, Mouse model, Reversal learning, Instrumental learning, Malaise, LiCl, Cognitive flexibility

## Abstract

Approximately 50–80 % of cancer patients suffer from cachexia, a metabolic syndrome involving inflammation, appetite loss, and muscle and fat wasting. Another common co-morbidity of cancer patients is cognitive impairment, and clinical evidence suggests the incidence of cachexia is linked to more severe cognitive symptoms. Given the difficulty of studying changes in cognitive function in human cancer patients, we set out to examine key aspects of cognitive performance in a mouse model of pancreatic cancer (pancreatic ductal adenocarcinoma; PDAC) cachexia, using an in-cage operant device (Feeding Experimental Device version 3; FED3) and a reversal learning task. Performance on the operant reversal task was compared to two control groups without cancer: *ad libitum* fed, sham injected with phosphate buffered saline (PBS), calorie restricted (CR) to 90–95 % of original body weight to control for reduced food intake and weight loss in cachexia mice. Our PDAC model recapitulated features of cachexia, including anorexia, weight loss, muscle wastage and inflammation. CR mice performed significantly better on the reversal task than both PDAC and PBS mice, achieving significantly more reversals and greater pellet retrieval. There was no difference between PBS and PDAC groups. These results suggest that the weight and appetite loss that occurs during cancer is processed by the brain differently to weight loss that occurs as a result of calorie restriction, with PDAC mice not experiencing an increase in motivational drive for food in line with their falling body weight. To mimic the malaise experienced by the PDAC group, we dosed CR mice with LiCl. Low dose (150 mM) LiCl did not affect responding, however, high dose (300 mM) LiCl significantly reduced both number of active pokes and pellet retrieval. This indicates a sickness-induced devaluation of reward, a factor that may impact poor performance of this task in the PDAC group. We additionally examined exploratory and anxiety-like behaviour in PBS and PDAC groups using a battery of maze-based tests. We saw no significant differences in performance between groups in the elevated plus maze, open field or light/dark box, suggesting no elevations in baseline anxiety-like symptoms in this cachexia model. These results occurred in the face of significantly elevated levels of the pro-cachexia factors GDF15, Activin A and Activin B, indicating that elevated levels of these TGF-β family peptides are not sufficient to produce behavioural changes in these tests. Our results provide evidence for a specific impact of sickness state on cognitive flexibility during pancreatic cancer.

## 1. Introduction

During acute sickness, behavioural and physiological responses, such as fever, hypophagia and fatigue, represent adaptive strategies to promote survival (Aubert, 1999). These processes, known as sickness behaviours, are regulated by inflammatory mediators and immune cells which communicate with the brain (Olson and Marks, 2019). Whilst beneficial in the short-term, ongoing inflammation during chronic conditions can result in long-term, deleterious changes to physiology and behaviour, and an initially adaptive strategy can become maladaptive, a type of executive dysfunction referred to as cognitive inflexibility.

Cancer cachexia is the involuntary weight loss that occurs secondary to cancer and is accompanied by fatigue, nausea, and physical frailty (Baracos et al., 2018). The symptoms of cachexia may be considered maladaptive sickness behaviours, for example the transition from short-term hypophagia to long-term anorexia (Olson and Marks, 2019). This ongoing inflammatory state has additional repercussions, with an increased incidence of a number of comorbidities including motivational deficits, mood changes and neurocognitive decline (Fortes et al., 2024).

In cancer, much of the literature around neurocognitive, mood and motivational impairment has focused on the impact of impairments occurring as a result of chemo- or radio-therapy treatment (Ahles et al., 2002; Dias-Carvalho et al., 2022; Tannock et al., 2004) There is, however, a developing literature indicating a direct impact of cancer on these processes. For example, cognitive impairment impacts approximately 30 % of patients before treatment, increasing to around 75 % during the active treatment phase. Up to 35 % of patients experience persistent loss of cognitive function, even after cancer treatment has concluded (Olson and Marks, 2019). This impact on cognition is present in the case of non-CNS tumours, with peripheral solid tumours able to induce cognitive decline (Jansen et al., 2011). There is additional evidence that the comorbidities of skeletal muscle loss and cognitive dysfunction are interconnected, as cancer patients suffering from cachexia show exacerbated depression (but not anxiety) symptoms, and reduced quality of life – a measure that includes social, emotional and functional wellbeing – even when time from diagnosis and cancer severity are controlled for (Nipp et al., 2018). In long term survivors of cancer, factor analysis groups the symptoms of cognitive functioning, fatigue, insomnia, pain, dyspnea, appetite loss, constipation, diarrhea, nausea and vomiting into a single cluster. This suggests a link between the typical symptoms of cachexia (appetite loss, nausea and vomiting, fatigue) and neurocognitive decline (Zucca et al., 2012), but it does not distinguish the differences between cancer-induced and cachexiainduced effects on mood and cognition, which could impede effective intervention strategies.

Disordered mood commonly occurs in those with cancer, with depression and anxiety repeatedly being identified as comorbid with metastatic cancer (Thavarajah et al., 2012). Elevated levels of anxiety in cancer patients are linked to poor nutritional status, a proxy measure of cachexia (Nardi et al., 2024). Changes in mood-related behaviour have been reported in mice in the Lewis lung carcinoma (LLC) model of cancer cachexia, where mice showed reduced exploratory behaviour in the open field and elevated plus maze tasks, as well as increased time to nest build (Campos et al., 2017). This likely reflects multiple aspects of mood and motivation-related changes induced by cancer and cachexia, including reduced locomotor activity, and increased fatigue, but whether these outcomes are more indicative of a motoric deficit rather than a motivational or cognitive deficit remains unknown.

Cognitive and motivational changes that occur during cachexia can be measured in mice using operant (reinforcement learning) tasks and apparatus such as the Feeding Experimental Device 3 (FED3) operant device have become increasingly recognised as a preferable alternative to traditional operant chambers. This is because these in-cage devices are placed into the home cage, allowing for reduced experimenter interaction and therefore reduced stress to the animals, as well as rapid task training times (Matikainen-Ankney et al., 2021). Mouse models allow the separation of mood and cognitive deficits that are present in human patients, in order to inform targeted interventions. One aspect of cognition with relevance to cancer cachexia is cognitive flexibility, which refers to the ability to switch between behavioural processes to adapt to a changing environment (Dajani and Uddin, 2015). There is considerable literature demonstrating cognitive flexibility is impacted in individuals with a history of cancer (Ahles and Root, 2018; Billiet et al., 2018; Kesler et al., 2013), however it is difficult to tease out the effects of cancer, cancer cachexia, and cancer treatment in human populations. Deficits in cognitive flexibility are also a hallmark of anorexia nervosa (Huang et al., 2023; Tchanturia et al., 2012), a condition of profound weight loss sharing clinical features with cachexia, namely the elevation of circulating proinflammatory cytokines [i.e. TGF-β, IL-6 and IL-1β; (Solmi et al., 2015)], and cognitive decline is also associated with lower muscle mass generally (Sui et al., 2021).

Mouse models of cancer cachexia exhibit fat and muscle loss, reduced appetite and elevated pro-cachexia factors. Models of a number of cancer types exist, but one of the most widely used is the KPC pancreatic cancer model. This model is an implantable form of syngeneic pancreatic cancer, originally derived from genetic modifications of the KRAS oncogene and TP52 tumour suppressor gene (Foley et al., 2015). It recapitulates the cardinal features of human pancreatic ductal adenocarcinoma (PDAC) (Michaelis et al., 2017) and is used widely in the field to investigate the pathophysiology of cachexia. This model has elevated levels of IL-6, IL-1β and TGF-β, all well-established pro-cachexia factors and markers of systemic inflammation (Arneson-Wissink et al., 2024; Grossberg et al., 2020; Michaelis et al., 2017). In order to test the impact of cancer with cachexia on cognitive function, we designed a reversal learning task to assess cognitive flexibility in mice using the FED3 devices. This task requires mice to adapt their behaviour to changing reward contingencies, reflective of flexible learning (Bissonette and Powell, 2012). We additionally used a series of maze-based tasks to probe mood-related behaviour and natural exploratory behaviour in mice with pancreatic cancer to parse out the potential differential effects of cognition and mood. We hypothesised that mice with cancer cachexia would exhibit a specific deficit in flexible learning above and beyond that associated with reduced food intake or body weight loss.

## Methods.

### 1.1. Animals

8–10-week-old C57Bl/6 male mice were obtained from Monash Animal Research Platform two weeks prior to treatment. Mice were allowed a week-long period of adaptation prior to the induction of pancreatic cancer, were single housed on a 12:12 light cycle with *ad libitum* access to standard mouse chow (Specialty Feeds, Glen Forrest, Western Australia) and tap water with nestlets and red Perspex shelters. Mice were regularly handled to reduce stress associated with investigator interactions. Male mice were used for this study as female mice are relatively resistant to cachexia in this model (Zhong et al., 2022), which mirrors the disease in humans (Zhong and Zimmers, 2020).

For FED3 reversal learning experiments, mice were allocated to 3 groups according to body weight to ensure equal distribution and variation in each group. The groups were PBS (sham injected with phosphate buffered saline) PDAC (pancreatic ductal adenocarcinoma cells suspended in PBS) and CR (calorie restricted). Each group initially consisted of eight mice, however three mice were excluded from analysis in the pancreatic ductal adenocarcinoma (PDAC) group due to no tumour (n = 2) or very small tumour (n = 1). Mice were housed as above, with the exception of the calorie restriction (CR) group, which received reduced food rations at the onset of the dark phase. Nightly rations were initially set at 2.7 g/mouse, and this was adjusted daily depending on weight change from the previous day with the goal of maintaining mice at 90 % of starting body weight. This value was chosen for two reasons, firstly it matches the final weight loss generally seen in the PDAC mice at time of sacrifice, and secondly, maintaining 90 % body weight is standard procedure in the field of motivational neuroscience for encouraging motivated responding in mice. Mice were exposed to sugar pellets 20 mg sucrose pellet (Able Scientific) several times in the week prior to the start of FED3 interactions to reduce neophobia. This approach has some limitations, as it cannot exactly match the metabolic changes experienced by PDAC mice (Arneson-Wissink et al., 2024), but was able to achieve matching of caloric intake over the final days of the experiment.

### 1.2. LiCl injections in CR mice

In order to induce subjective feelings of malaise in the CR mice, they were dosed with Lithium Chloride (LiCl), well established to induce sickness behaviours. On Day 10 and 11, CR mice received an intraperitoneal injection (IP) of low dose LiCl (150 mM given at 10 ml/kg). Phosphate buffered saline (PBS; vehicle control) and PDAC mice received an intraperitoneal injection of saline (10 ml/kg). On Day 13, the dose of LiCl was increased to 300 mM given at 10 ml/kg (high dose). A dose of 150 mM is sufficient to induce a mild conditioned taste aversion in our hands (data not shown), but does not suppress food intake in overnight fasted mice (equivalent to 1 % dose in (Lachey et al., 2005)) while 300 mM will cause a robust taste aversion in the conditioned taste preference test in our hands (data not shown) and significantly suppresses refeeding in overnight fasted mice (equivalent to 2 % dose (Lachey et al., 2005)). All injections were administered 1 h before reversal task testing.

### 1.3. Tissue culture

PDAC (KPC) cells were generously gifted by Daniel Marks (Oregon Health & Science University, Portland, OR, USA), originally isolated as per (Foley et al., 2015). PDAC cells were cultured at 37 °C in flasks containing 40 ml of culture media and 400 μl of Penicillin Streptomycin solution (Gibco). Media was prepared as previously described (Michaelis et al., 2017): 88 % Rosewell Park Memorial Institute (RPMI) 1640 media, 10 % heat inactivated fetal bovine serum (Gibco), 1 % 1 mM sodium pyruvate (Gibco) and 1 % Minimum Essential Medium Non-Essential Amino Acids (Gibco).

For all injections into mice, PDAC cells were defrosted and passaged once, expanded and then prepared for injection to ensure no passage effects could occur. To prepare cells, media was removed, cells were washed with PBS, then incubated in 3x trypsin enzyme (Gibco, #R001100) for 5 min at 37 °C. Cells were then collected with 10 ml of media to neutralise trypsin, then centrifuged for 5 min at 1000 rpm. The cells were then resuspended in 10 ml fresh media counted using an automatic cell counter (Luna 3, Logos Biosystems). For injection, cells were resuspended in PBS at a density of 5×10^5^/300ul. Mice were briefly anaesthetised using isoflurane (2 % in oxygen) and received an IP injection of either 300 μl PDAC cells or PBS (sham controls). Mice were injected using a 25-gauge needle attached to a 1 ml syringe directed at the lower left side. Day of PDAC injection is considered day 0.

### 1.4. Behaviour tests

#### 1.4.1. FED3 training and reversal learning task

Three days prior to injection of PDAC cells, mice were exposed to the FED3s on a fixed ratio 1 (FR1) with pellet delivery occurring in response to pokes into both left and right nose poke ports to habituate them to the devices. Testing was performed in 2×90min epochs each day, during the mid-light phase, with an equal number of mice from each treatment group in each. On Day 1 and 2 of FR1 both nose-poke ports were active (FR1 Both) to assess baseline responding (total pellets obtained). Three low responders (0 to 4 pellets per day) were identified and these mice were evenly distributed across groups. We also assessed side bias for nose poke ports in order to control for side bias in the reversal task. A side bias was characterised by > 65 % of total pokes being on one side (left or right).

The reversal task required 3 consecutive correct nose pokes into the ‘target’ (i.e., active) port of the FED3, resulting in delivery of a 20 mg sucrose pellet (Able Scientific). The contingencies then reversed, with the previously inactive port switching to become the ‘target’. Mice were required to guide their action selection accordingly to obtain the most rewards. The rewarded action continuously alternated in a within-session serial reversing manner, throughout the 90-minute testing period. A reversal criterion of three correct actions before reversal was chosen to balance the ability to observe a higher number of reversals with the maximum number of pellets mice are able to consume in a session. Previous observations from FR1 both training demonstrated that non-calorie restricted mice poked for 8–15 pellets per session.

Mice were tested on the reversal task on days 3 and 4 following pancreatic cancer cell injection (PDAC group), and the start of food restriction (CR group). At this time point no clinical signs of cancer are present, and tumours are not detectable at necropsy (data not shown). Mice were then tested again on the reversal task on Days 7–11 and 13. On all other days, mice did not have access to FED3s. CR mice were dosed with Lithium Chloride (LiCl) on Day 10, 11 (150 mM at 10 ml/kg) and 13 (300 mM at 10 ml/kg). This protocol controls for malaise in PDAC mice resulting in poor performance, with the increasing dose matching increasing illness in the PDAC group. All injections were administered 1 h before reversal task testing. FED3 data was processed through a custom Python code (available at https://github.com/Foldi-Lab/LKM_FED3-tasks).

#### 1.4.2. Light/Dark boxes

In a separate cohort of mice, we performed a series of maze-based tests. In this experiment, we only used PDAC and PBS groups. Eight days after cancer was induced via injection of PDAC cells (cancer induction), animals were individually placed in the dark side of the apparatus and the top covered. The light/dark box apparatus consisted of a two-chambered box, with a large, white zone (480 × 300 mm) and a smaller black zone (150 × 300 mm). The white zone was open to the light, and the dark zone was covered. Mice have free access to both spaces. Mice were then allowed to explore the space for six minutes. Mouse order alternated between control and cancer groups.

#### 1.4.3. Open field

Ten days after cancer induction, animals were individually placed in a side of the field and allowed to explore for six minutes. The open field (OF) test consisted of an 800 mm in diameter round field surrounded by walls in which distance travelled in each 6-min trial was used as the primary measure of locomotor activity and the proportion of time spent in the aversive centre zone (~middle third, by diameter; middle zone circumference defined as 280 mm from wall) was used as a secondary measure of anxiety-related behaviour, although it is shown to be less sensitive to the effects of anxiolytic drugs. Mice alternated between control and cancer groups.

#### 1.4.4. Elevated plus Maze

Twelve days after cancer induction (or control injection), animals were individually placed in the neutral centre of the maze and allowed to explore for six minutes. The elevated plus maze (EPM) consisted of an elevated 4-arm platform made of grey Perspex (with each arm being 50 × 300 mm, and a centre zone 50 × 50 mm), with two enclosed (250 mm high walls) and two open arms. Mice were placed in the centre platform facing an open arm and the proportion of time spent in the closed arms relative to the open arms in each 6-min trial was used as the primary measure of anxiety-like behaviour. Mice alternated between control and cancer group.

#### 1.4.5. Video analysis

All behavioural tests were recorded with an overhead camera and later scored on a PC computer using Ethovision XT software (Noldus, Netherlands). Mice were scored for time in zone (task-specific zones as per (Lockie et al., 2017)), velocity and distance travelled.

#### 1.4.6. Non-food consummatory behaviours

To control for the possibility that lack of feeding in PDAC mice was related to other behavioural phenotypes, we assessed time spent gnawing wooden dowel and kaolin, both indigestible and non-nutritive substances that may indicate signs of compulsive behaviour (dowel gnawing) or to alleviate malaise (kaolin). A pellet of kaolin or dowel was placed into each cage overnight and the extent to which animals consumed these substances was recorded.

#### 1.4.7. ELISA for GDF15, Activin A and Activin B

GDF15 ELISA was performed as per manufacturers instructions (Mouse GDF15 DuoSet ELISA, DY6385, R&D systems). Activin B ELISA was performed as per manufactures instruction (ActivinB ELISA AL-150, Ansh Labs). Activin A ELISA had serum samples diluted 1:5 in 1 % bovine serum albumin (BSA) and analysed as previously described (Chen et al., 2014).

## 2. Results

### 2.1. Feeding behaviour and body composition

**Fig. 1.**
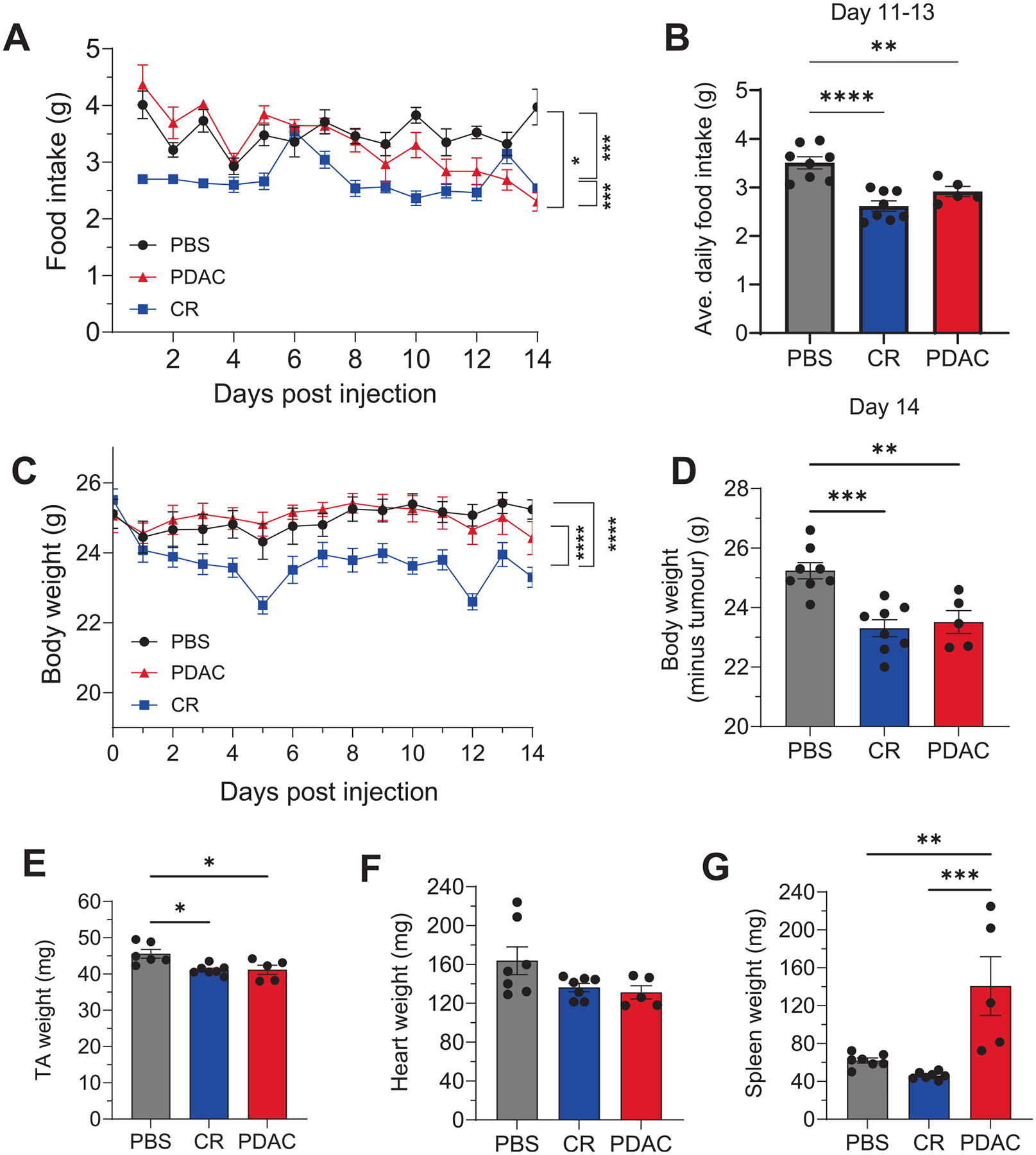
Pancreatic cancer and calorie restriction result in a similar phenotype with respect to body weight and muscle loss. (A) Food intake was significantly different between groups across the 14 days of the experiment (PBS vs CR *p* < 0.0001; PBS vs PDAC *p* = 0.0304, RM ANOVA). Food available to CR mice was dynamically changed each day to ensure weight loss to approximately 90 % of free feeding body weight. (B) Food intake for each group, averaged across the final three days of the experiment (days 11–13) (PBS vs CR *p* = 0.0088; PBS vs PDAC *p* < 0.0001, one way ANOVA with Tukey’s post-hoc test). (C) Body weight of each group across the 14 days of the experiment, weight changes were monitored daily to ensure CR mice maintained ~ 90 % of free feeding body weight (PBS vs CR *p* < 0.0001; CR vs PDAC *p* = 0.0001, RM ANOVA). (D) Body weight minus tumour weight (for PDAC group) at necropsy demonstrates excellent matching for CR and PDAC groups (PBS vs CR *p* = 0.0004; PBS vs PDAC *p* = 0.0039, one way ANOVA with Tukey’s post-hoc test). (E) Tibialis anterior weight was taken at necropsy as a measure of skeletal muscle wasting, with both PDAC and CR groups showing significantly reduced muscle weights (PBS vs CR *p* = 0.0115; PBS vs PDAC *p* = 0.0214, one way ANOVA with Tukey’s post-hoc test). (F) Heart weight was also taken at necropsy, as a measure of cardiac muscle wasting, which was non-significantly lighter in the PDAC and CR groups (PBS vs CR *p* = 0.1358; PBS vs PDAC *p* = 0.1035, one way ANOVA with Tukey’s post-hoc test). (G) spleen weight at necropsy, as a proxy measure for inflammatory state, was significantly heavier in the PDAC group compared to both CR and PBS (PDAC vs CR *p* = 0.0008; PBS vs PDAC *p* = 0.0040, one way ANOVA with Tukey’s post-hoc test). *p < 0.05, **p < 0.01, ***p < 0.001,****p < 0.0001, compared to PBS;

In order to generate a calorie restricted control group that was not different in body weight to the PDAC group at endpoint, we dynamically restricted food intake in the CR group to approximately 25 % of the *ad lib* fed, PBS treated group (PBS). This resulted in significantly reduced food intake in both the CR and PDAC groups, when compared to *ad lib* fed mice (PBS vs CR *p* < 0.0001; PBS vs PDAC *p* = 0.0304, RM ANOVA) (Fig. 1A). This resulted in average food intake over the final three days of the experiment (11–13, mice killed on the morning of day 14) that was not different between the CR and PDAC groups, and both groups had significantly reduced food intake compared to ad lib fed mice (PBS vs CR *p* = 0.0088; PBS vs PDAC *p* < 0.0001, one way ANOVA with Tukey’s post-hoc test) (Fig. 1B). This CR protocol produced a body weight reduction of 5–10 % compared to baseline in the CR group, resulting in significantly lower weights in the CR and PDAC groups when compared to the PBS group (PBS vs CR *p* < 0.0001; PBS vs PDAC *p* = 0.0304, RM ANOVA) (Fig. 1C). Importantly, PDAC bearing mice grow sizable tumours (tumour weight average was 919.4 mg, ±120.6 (SEM)) and the body weight data in Fig. 1C does not take this into account. When tumour-free mass was plotted after necropsy, body weight was not different between PDAC-bearing and CR mice (*p* > 0.05), and both were significantly lighter than *ad lib* fed mice (PBS vs CR *p* = 0.0088; PBS vs PDAC *p* < 0.0001, one way ANOVA with Tukey’s post-hoc test). Body weight for both groups was significantly lower than *ad lib* fed mice (PBS vs CR *p* = 0.0004; PBS vs PDAC *p* = 0.0039, one way ANOVA with Tukey’s post-hoc test) (Fig. 1D). As an additional measure of body composition, weight of tibialis anterior, as a representative skeletal muscle, were significantly lighter in both PDAC and CR groups, compared to *ad lib* fed mice (PBS vs CR *p* = 0.0115; PBS vs PDAC *p* = 0.0214, one way ANOVA with Tukey’s post-hoc test) (Fig. 1E). Heart weights were reduced in CR and PDAC groups, but not significantly so (PBS vs CR *p* > 0.05; PBS vs PDAC *p* > 0.05, one way ANOVA with Tukey’s post-hoc test) (Fig. 1F). Spleen weight was significantly higher in the PDAC group compared to both other groups (PBS vs CR *p* = 0.0008; PBS vs PDAC *p* = 0.0040, one way ANOVA with Tukey’s post-hoc test) (Fig. 1G), indicative of the broad inflammatory state of this group.

### 2.2. Cognitive performance

**Fig. 2.**
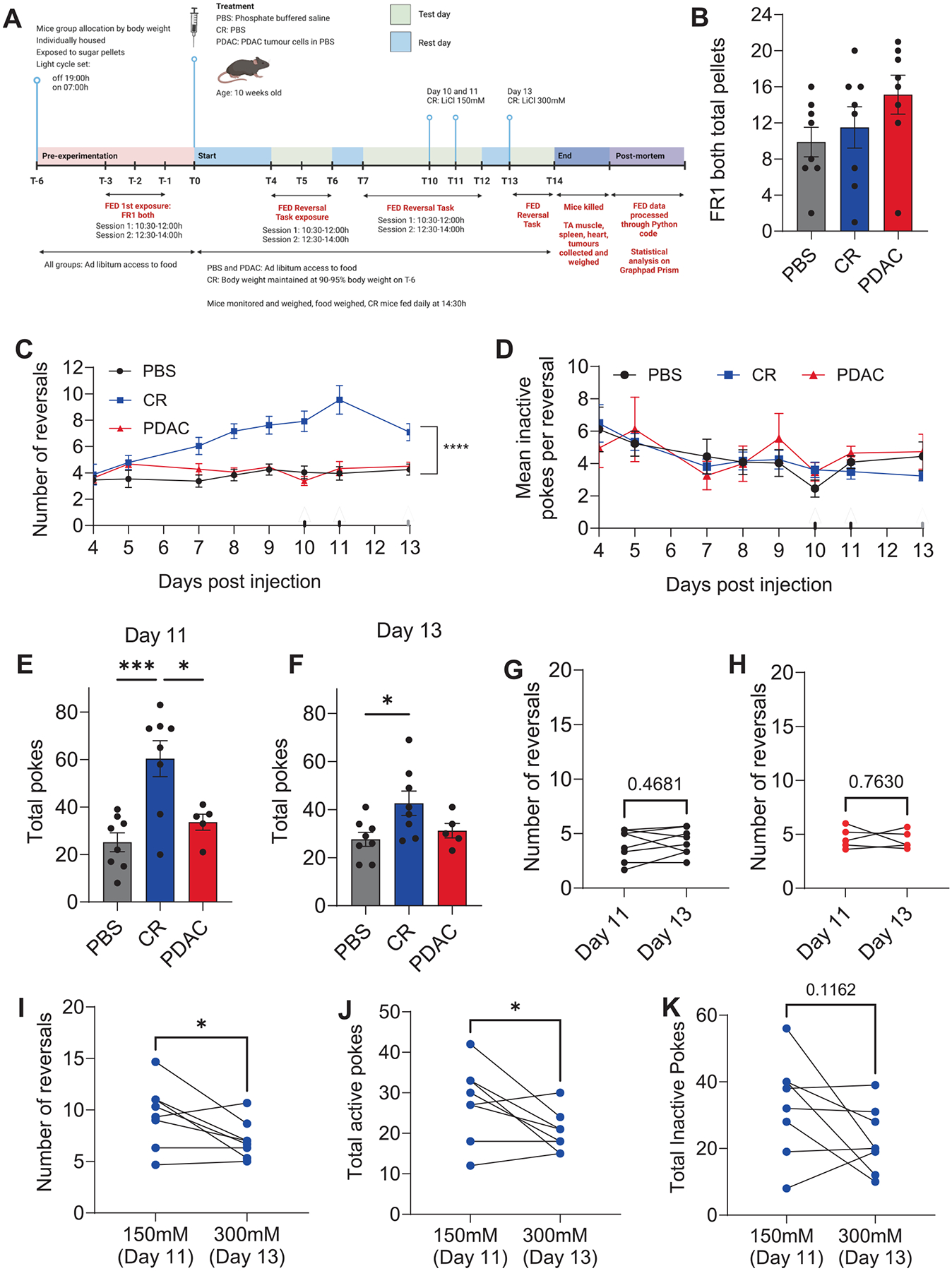
Cachexia and LiCl-induced malaise impacts instrumental learning, but not contingency learning. (A) Schematic of operant learning experimental time course. (B) Prior to induction of cancer, or calorie restriction, all groups performed similarly on the fixed ratio 1 operant task (*p* > 0.05, one way ANOVA). (C) Number of reversals was significantly greater in CR mice compared to PBS treated, or PDAC-bearing mice. Black arrows indicate administration of 150 mM LiCl (10 ml/kg), grey arrow indicates 300 mM LiCl administration (10 ml/kg). (PBS vs CR *p* < 0.0001; CR vs PDAC *p* < 0.0001, RM ANOVA, Šídák’s multiple comparisons test). (D) Mean inactive pokes per reversal is a measure of task accuracy, and was not different between groups across time (RM ANOVA, *p* > 0.05). (E) On day 11 of cachexia, total pokes (active + inactive) was significantly higher in the CR group than both the PBS and PDAC groups, in spite of administration of 150 mM of LiCl to the CR group to control for malaise on this day (PBS vs CR *p* = 0.0319; PBS vs PDAC *p* = 0.1811, one way ANOVA with Tukey’s post-hoc test). (F) On day 13 of cachexia, the CR group made significantly more total pokes than the PBS group, but was not significantly different to the PDAC group, suggesting an impact of 300 mM LiCl on responding (PBS vs CR *p* = 0.0319; PBS vs PDAC *p* = 0.1811, one way ANOVA with Tukey’s post-hoc test). Number of reversals was not different in either PBS (*p* = 0.4681, paired *t*-test) (G) or PDAC (*p* = 0.7630, paired *t*-test) groups (H) between days 11 and 13, however it was significantly reduced in the CR group (*p* = 0.0432, paired *t*-test) (I), indicating a significant impact of LiCl-induced malaise on instrumental responding in this group. This was driven by significantly decreased active pokes (*p* = 0.464, paired *t*-test) (J), as there was no significant difference in inactive pokes (*p* = 0.1162, paired *t*-test) (K). *p < 0.05, ***p < 0.001,****p < 0.0001, compared to PBS or PDAC.

To assess cognitive flexibility and motivated responding, we used the FED3s in the home cage (Fig. 2A). Mice were initially trained on fixed ratio 1, and responding was not different across groups (*p* > 0.05, one way ANOVA) (Fig. 2B). Mice performed the reversal task (FR3) from day 4 of the experiment onwards.

The number of reversals was not different between groups at days 4 and 5, however by day 7 the CR group was completing significantly more reversals than both the PBS and PDAC groups (PBS vs CR *p* < 0.0001; CR vs PDAC *p* < 0.0001, RM ANOVA, Šídák’s multiple comparisons test) (Fig. 2C). The number of completed reversals continued to escalate over the course of the experiment, i.e. depicting a learning curve for the CR group, similar in extent to what is seen in other operant tasks that involve a level of CR to motivate performance, and increased familiarity with the task (Fig. 2C). Responding was not reduced by 150 mM LiCl injection, delivered on days 10 and 11 to mimic malaise associated with cancer (Fig. 2C, black arrows). On day 13, 300 mM LiCl was delivered to mimic increasing illness in the PDAC group. This slightly dampened responding, although the CR group still performed more reversals than the other two groups (Fig. 2C, grey arrow). In spite of falling body weight and food intake (Fig. 1A and B), PDAC-bearing mice never increased their reversals above baseline, remaining similar to PBS (Fig. 2C).

Given that the reversal task is designed to assess cognitive flexibility, and cancer is associated with cognitive impairment, we compared nose pokes into the active and inactive ports in PDAC bearing mice. Excessive pokes into the inactive port is indicative of failure to learn or remember the task, and this could explain the inability of PDAC-bearing mice to increase responding with reducing food intake and body weight. Mean inactive pokes per reversal was not different between groups across time (*p* > 0.05, RM ANOVA) (Fig. 2D), indicating that neither progression of pancreatic cancer, nor LiCl treatment in CR mice impacts accuracy in this task.

Total pokes can be interpreted as a proxy for engagement with, and motivation for, the task. On both days 11 (PBS vs CR *p* = 0.0007; PBS vs PDAC *p* = 0.0188, one way ANOVA with Tukey’s post-hoc test) (Fig. 2E) and 13 (PBS vs CR *p* = 0.0319; PBS vs PDAC *p* = 0.1811, one way ANOVA with Tukey’s post-hoc test), the CR group had the greatest number of total pokes. Engagement from this group was highest on day 11 (Fig. 2E), in spite of administration of low dose LiCl, which suggests that regardless of low dose LiCl administration, CR mice were still exhibiting a learning curve over time with task proficiency increasing until this point. However, administration of higher dose LiCl on Day 13 tended to reduce total pokes in this group (Fig. 2F). Given this strong downward trend in total pokes between day 11 and 13 in CR mice administered with LiCl, we compared reversals achieved by each group between these days. There was no difference in number of reversals between PBS (*p* = 0.4681, paired *t*-test) or PDAC (*p* = 0.7630, paired *t*-test) groups across these two days (Figs. 2G and 2H). However, the CR LiCl group showed a significant reduction in the number of reversals achieved with high dose (day 13) compared to low dose (day 11) LiCl (*p* = 0.0432, paired *t*-test) (Fig. 2I). This was mirrored by a drop in total active pokes, as expected (*p* = 0.4640, paired *t*-test) (Fig. 2J). Inactive pokes were not significantly lower on day 13 compared to day 11 (Fig. 2K). This decrease in performance at the higher dose of LiCl (300 mM) indicates that LiCl not only disrupted the expected learning curve but likely altered the value of reward and so reduced action selection at the active poke specifically. Taken together, this suggests that the level of sickness induced can alter the ability to make appropriate action selections and hinder cognitive flexibility, even in a highly food-motivated state.

### 2.3. Mood-related behaviour

**Fig. 3.**
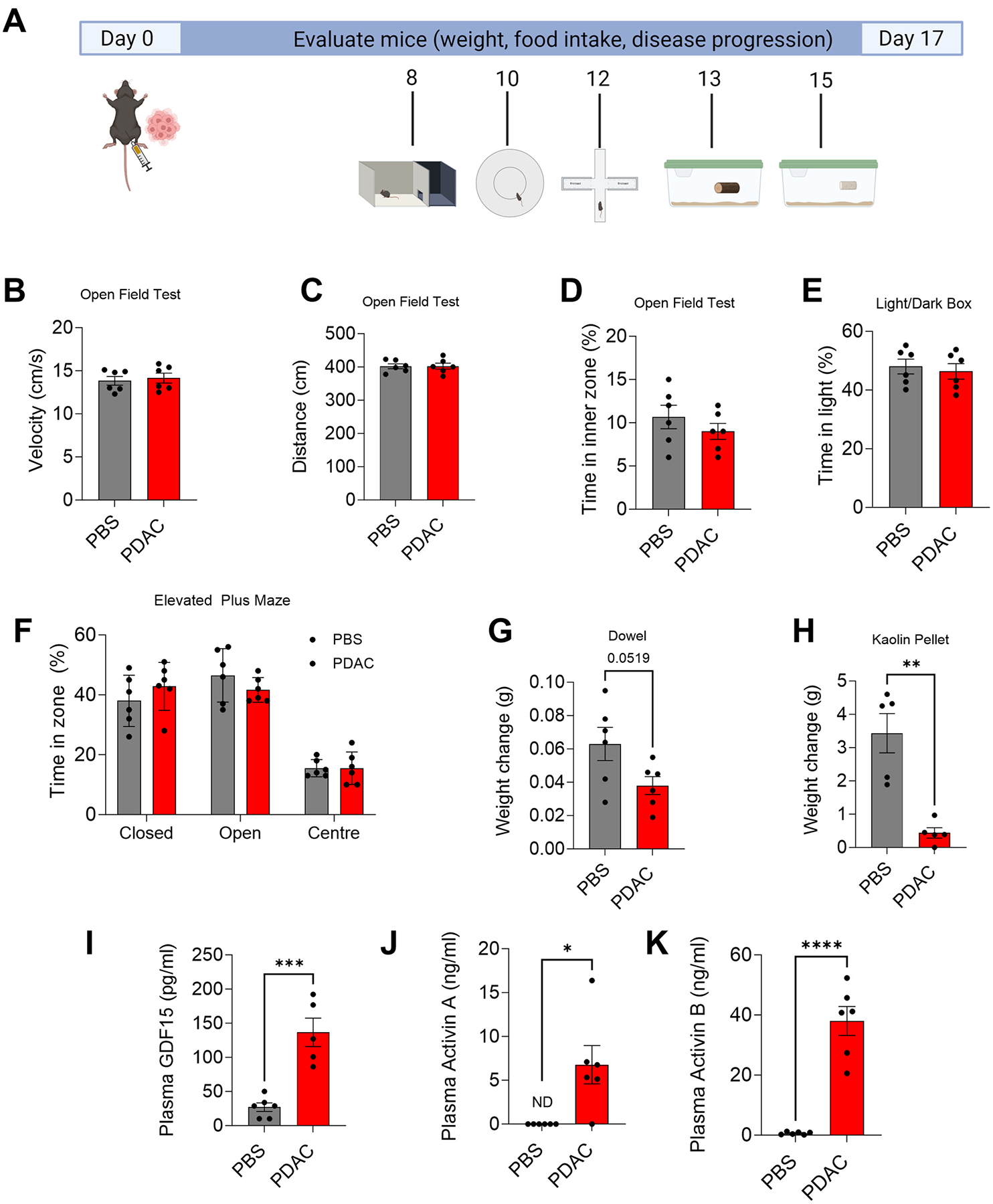
PDAC associated cachexia does not alter mood-related behaviour. (A) Schematic of operant learning experimental time course. In the Open Field Test, no differences were seen in (B) velocity (*p* = 0.6876), (C) distance travelled (*p* = 0.9887), or (D) percent time in inner zone (*p* = 0.6876, all unpaired *t*-test). (E) Percent time in the light side of the light/dark box was not different between control and PDAC-bearing mice (*p* = 0.6582, unpaired *t*-test). (F) No differences were seen between control and PDAC-bearing mice in percent time spent in any zone of the elevated plus maze (*p* = 0.2560, ANOVA). There was reduced non-nutritive consumption of (G) wooden dowel (non-significant; *p* = 0.0519, unpaired *t*-test) and (H) kaolin pellets by mice with PDAC (*p* = 0.0012, unpaired *t*-test), **p < 0.01. Plasma levels of (I) GDF-15 (*p* = 0.0004, unpaired *t* test), (J) Activin A (*p* = 0.0111, unpaired *t* test) and (K) Activin B (*p* < 0.0001, unpaired *t* test) were all significantly elevated following sacrifice on day 15. *p < 0.05; **p < 0.01; ***p < 0.001; ****p < 0.0001.

In a separate cohort of mice, we set out to examine the impact of pancreatic cancer and the accompanying cachexia on exploratory activity in a battery of behavioural tests. This battery has traditionally been used to examine anxiety-like behaviour and locomotor behaviour, but performance in these tests can also inform aspects of motivation and risk-taking behaviour. We performed three complementary tests on days 8–12 of our cancer protocol (Fig. 1A). This period equates to mild cachexia, with the onset of anorexia usually occurring on day 9–10. We chose this period to ensure mice did not experience severe locomotor impairment due to cachexia, a known confound of this kind of test. We saw no differences in either velocity or distance (*p* = 0.6876, unpaired *t*-test) or (*p* = 0.9887, unpaired *t*-test) in the Open field (Figs. 3B and 3C), indicating there is no deficit in locomotion in the PDAC-bearing mice during mild cachexia. In the three tests of anxiety, we saw no difference in time spent in the inner zone in the open field test (*p* = 0.6876, unpaired *t*-test) (Fig. 3D), time spent in the light zone in the light/dark box (*p* = 0.6582, unpaired *t*-test) (Fig. 3E) or time spent in the open arms of the elevated plus maze (*p* = 0.2560, unpaired *t*-test) (Fig. 3F). Together, this indicates no impact of pancreatic cancer, or mid-stage cachexia, on anxiety-like behaviour or natural exploratory behaviour in this mouse model. Pica is the consumption of non-food items, and in rodents that lack the capacity to vomit, it serves as a way of self-soothing nausea (Yamamoto et al., 2002). We used both wooden dowels and kaolin clay pellets to assess pica in mice with pancreatic cancer cachexia. In both cases, PDAC-bearing mice gnawed less than control mice, with this being a not quite significant effect for dowels (Fig. 3G) (*p* = 0.0519, unpaired *t*-test), but quite significant for kaolin pellets (*p* = 0.0012, unpaired *t*-test) (Fig. 3H).

Given cachexia is associated with elevated levels of circulating TGF-β ligands (Chen et al., 2016), we measured plasma levels of GDF-15, Activin A and Activin B following sacrifice on day 15. We saw significantly elevated levels of GDF15 (Fig. 3I, p = 0.0004, unpaired *t* test), Activin A (Fig. 3J, p = 0.0111, unpaired *t* test) and Activin B (Fig. 3K, p < 0.0001, unpaired *t* test).

## 3. Discussion

Using a within-session serial reversal learning task with deterministic reward contingencies, we assessed the ability of mice with pancreatic cancer to adapt behaviour to a dynamically changing environment, and compared performance to wild-type mice that were exposed to either *ad libitum* food access or matched calorie restricted conditions. Animals from all groups displayed an ability for instrumental learning (i.e. nose-poking into the FED3 device port will deliver sucrose reward pellets) during the initial training period (FR1 Both) with no significant differences in the number of rewards earned. Although the number of completed reversals, which is indicative of cognitive flexibility during the serial reversal learning period of testing, was not different between the mice with pancreatic cancer and the *ad libitum* fed mice, calorie restricted mice performed significantly more reversals than both the other groups, with the separation occurring from day 7 onwards. While calorie restricted mice showed the expected learning curve throughout task acquisition, neither *ad libitum* fed mice or mice with pancreatic cancer displayed an ability to increase task proficiency over time beyond the initial 3 reversals, with performance plateauing from the first day of testing. Additionally, the behavioural test battery conducted in a separate cohort of mice revealed that the lack of improvement in reversal learning in mice with pancreatic cancer was not due to impairments in motor function or increased anxiety-like behaviour. In accordance with this, a reduction in nausea-induced self-soothing behaviours in mice with pancreatic cancer suggests that behaviour was not directed away from performing the task. Seemingly, the reversal learning impairment in mice with pancreatic cancer is likely due to a specific sickness-induced devaluation of sucrose reward that impairs correct action selection and subsequent ability to adapt behaviour appropriately since LiCl dose-dependently altered performance.

Calorie restriction is commonly used to promote task engagement through increasing incentive motivation and improving attentional performance, so this improvement in reversal learning over time in comparison to *ad lib* fed (PBS) mice is unsurprising. Since cachexia results in reduced appetite and subsequent body weight loss, it was necessary to have a weight matched calorie restricted group. At end point, the mice with pancreatic cancer had similar average daily food intake, cancer free body weight, and tibialis anterior weight (as a proxy for skeletal muscle mass) to the calorie restricted mice. With food intake and body weight loss controlled for, we unmasked a significant reduction in reversals performed in the pancreatic cancer group. Importantly, this does not appear to be due to an inability to understand the task, as the tendency to poke into the inactive port was not higher in this group, but rather appears to be a deficit in motivation or engagement with the task. This suggests that the loss of body weight that occurs secondary to cancer is processed by the brain in a fundamentally different way to that which occurs as a result of calorie restriction with respect to motivational drive. Alternatively, these outcomes could indicate that the increase in motivational drive observed during calorie restriction is blunted due to cancer, which could be independent of body weight loss.

The behavioural response to illness impacts a range of domains including motivation (Der-Avakian et al., 2016). It is reasonable to expect that malaise experienced with cancer would impact performance in our reversal learning task. To address this, we dosed mice in the calorie restricted group with increasing doses of LiCl on days 10, 11, and 13. The doses were chosen to mimic mild illness on days 10 and 11 (the point in pancreatic cancer progression where we see divergence in food intake) and more severe illness on day 13, when we see clearly suppressed food intake and body weight loss in mice with pancreatic cancer. Low dose LiCl did not impact responding, however when given at a dose capable of suppressing food intake in fasted mice (300 mM), we observed significantly reduced active responses and thus reversals achieved. Indeed, others have demonstrated devaluation of rewarded levers following pairing of the active lever with LiCl with 300 mM of LiCl, but not at 150 mM of LiCl in food restricted rats (Paredes-Olay and Lopez, 2002). Critically though, even this dose resulted in significantly higher responding in the calorie restricted group compared to the pancreatic cancer group, suggesting that the impairment in valued responding is due to more than subjective feelings of malaise in the PDAC-bearing mice. Calorie restriction normally heightens motivation for reward, which is why CR controls showed strong engagement and the expected improvement in reversal performance with experience. When LiCl was administered to CR mice, performance declined despite this increased appetite, demonstrating that sickness can specifically disrupt reward valuation and flexibility. PDAC mice, however, failed to show learning improvements across sessions even though their overall task engagement was comparable to fully-fed controls and their food intake resembled that of CR mice. This pattern indicates that reduced appetite or satiety alone cannot explain their deficits. Instead, PDAC likely produces a distinct impairment in reward valuation and cognitive flexibility that emerges despite intact motivation to work for reward.

The maze-based tasks used here have traditionally been used to assess anxiety-like behaviour, and have the advantage of engaging natural aspects of rodent behaviour, in a way that operant responding tasks cannot. Increases in anxiety-like behaviour has been reported in the LLC model of cachexia (Campos et al., 2017), but we saw no such effect here in any of the three tests we used. Others have shown decreased voluntary activity in cancer models – decreased wheel running, increased immobility in the Porsolt forced swim test and decreased grip strength (Norden et al., 2015), alongside reduced consumption of sucrose and interpreted it as increased depressive mood. Here, we show, via a specific reduction in “active” (reward-related) nose-poking, that PDAC-bearing mice lose the ability to value the rewarding properties of sugar, without any specific changes in mood-related behaviours.

We did not test the effect of LiCl-induced malaise on performance in the maze tasks in this study, but others have demonstrated the effect of LiCl in wild-type mice, LiCl dose-dependently reduces total distance travelled in an open-field, as well as the time spent in the open arms of an elevated plus maze. This points to subjective feelings of malaise impacting the expression of natural rodent behaviours, which likely reflects a complex interplay between processes of reduced motivation to seek appetitive stimuli and increased avoidance behaviour (Heinz et al., 2021).

Reduced sucrose preference or consumption in 2-bottle choice tasks has been seen in a number of rodent models of cancer (Lamkin et al., 2011; Nashed et al., 2015; Norden et al., 2015; Pyter et al., 2009) and has been widely interpreted as a depressive marker, especially when seen alongside increased immobility time in the forced swim test. The validity of the forced swim test for assessing ‘depression-like’ behaviour is now being widely questioned (Molendijk and de Kloet, 2019), and it has dropped out of favour in many jurisdictions. Reduced sucrose consumption is also a sign of reduced motivation and of reduced hedonic processing. The impact of cancer on both these processes is unclear, with no effect on immobility time in the forced swim test or on sucrose consumption seen in mouse models of ovarian cancer (Chen et al., 2024; Lamkin et al., 2011), but increased anxiety-like behaviour in rats with breast cancer (Pyter et al., 2009). However, considering that PDAC-bearing mice in the present study poked for sucrose pellets at the same rate during training as *ad libitum* fed controls, our results do not support a specific influence of cancer cachexia on depression-like behaviours. We also assessed dowel gnawing and kaolin consumption to investigate the presence of pica. Contrary to our hypothesis, we saw significantly reduced consumption of kaolin in the PDAC-bearing group, indicating they were not experiencing ‘classical’ sickness behaviours, along with nearly significantly reduced gnawing of dowels. Dowel gnawing is a recognised stereotypic behaviour induced by excess striatal dopamine (Ernst, 1967), perhaps suggesting a motoric dopaminergic impairment that was specific for consumption, as it did not translate to changes in locomotor activity in the maze-based tests.

Inflammation is a described driver of change in behavioural tests, and elevated levels of IL-6, IL-1β and TGF-β are present in this model (Arneson-Wissink et al., 2024; Grossberg et al., 2020; Marks et al., 2001). We have additionally shown that GDF15, Activin A and Activin B are also elevated. These factors contribute to muscle wasting in cachexia (Chen et al., 2017; Groarke et al., 2024) but our results indicate they do not have a significant impact on maze-based tests. We did not directly test whether elevated levels of GDF15 or activins caused the observed deficits in performance on the cognitive task, however GDF15 has been recently implicated in cognitive dysfunction, with plasma levels correlating with cognitive impairment in sepsis and neurodegenerative conditions (Chen et al., 2025; Kochlik et al., 2024), so there could plausibly be a role for elevated GDF15 the deficits described in this paper.

Perhaps examining simple rodent sickness behaviours through the above-mentioned tests do not reveal the nuances of cancer cachexia on functional outcomes (e.g. cognitive flexibility). Here we present reversal learning as a more sensitive cognitive-behavioural paradigm to model the detrimental effects of sickness in cancer cachexia. Others have demonstrated cognitive deficits in mouse models of cancer, although not specifically reversal learning. In a model of breast cancer, mice with cancer demonstrated reduced interaction with the novel object-location in a combined novel object/novel place recognition task, indicating reduced capacity to remember the familiar object-location. This deficit occurred prior to advanced disease or locomotor effects that may indicate fatigue. Breast cancer is not a typically cachectic cancer, and the mice in this study only lost body weight at the very end of the study, well after the memory testing (Walker et al., 2018). In line with evidence in rats, an illness induced state that profoundly affects food consumption (like cancer cachexia), changes sensory cortical responses to tastes (Stone et al., 2022). We show here that it is this specific interaction of cancer with a sickness state that results in impairments in cognitive flexibility. This type of cognitive impairment can destabilize awareness of optimal choice behaviour and reinforces perseveration on disadvan-tageous ones, affecting functional outcomes in patients across a range of neurological and psychiatric conditions (Luo et al., 2024; Mantonakis et al., 2024). This data may go on to inform the management of sickness in cachectic cancer patients to improve functional outcomes and quality of life.

## Data Availability

Data will be made available on request.
